# Gender-Specific Effect of -102G>A Polymorphism in Insulin Induced Gene 2 on Obesity in Chinese Children

**DOI:** 10.1155/2015/872506

**Published:** 2015-06-16

**Authors:** Fang-Hong Liu, Jie-Yun Song, Yi-Ning Zhang, Jun Ma, Hai-Jun Wang

**Affiliations:** Institute of Child and Adolescent Health, School of Public Health, Peking University, Beijing 100191, China

## Abstract

*Background.* Insulin induced gene 2 (*INSIG2*) encodes a protein that has a biological effect on regulation of adipocyte metabolism and body weight. This study aimed to investigate the association of *INSIG2* gene -102G>A polymorphism with obesity related phenotypes in Chinese children and test gender-specific effects. *Methods.* The 2,030 independent individuals aged from 7 to 18 years, including 705 obese cases and 1,325 nonobese controls, were recruited from local schools. We measured the obesity-related phenotypes and detected the serum lipids. We genotype -102G>A polymorphism by using the matrix-assisted laser desorption/ionization time-of-flight mass spectrometry (MALDI-TOF MS). *Results.* In all individuals, we found that the GG/GA genotype of *INSIG2* -102G>A polymorphism was associated with risk of severe obesity (OR = 1.62, 95% CI: 1.11–2.36, and *P* = 0.012) under the dominant model. The association with severe obesity existed only in boys (OR = 1.91, 95% CI: 1.15–3.17, *P* = 0.012). The GG/GA genotype of -102G>A polymorphism was also associated with higher waist circumference (*β* = 2.61 cm, *P* = 0.031) in boys. No similar association was found in girls. The polymorphism was not associated with other obesity-related phenotypes, neither in all individuals nor in gender-specific population. *Conclusions.* This study identified a gender-specific effect of *INSIG2* -102G>A polymorphism on risk of severe obesity and waist circumference in Chinese boys.

## 1. Introduction

Obesity has been associated with many common medical conditions, such as type 2 diabetes, hypertension, cardiovascular disease, stroke, and physical disabilities [1]. The prevalence of obesity has been increasing quickly in the world, not only in developed countries but also in developing countries, being a serious public health problem.

Obesity has familial predisposition and high heredity, indicating the role of genetic factors in pathogenesis of obesity. Herbert et al. [2] reported that a common variation (rs7566605) located 10 kb upstream of insulin induced gene 2 (*INSIG2*) was associated with obesity in the first obesity genome-wide association study (GWAS) in 2006. Since then, association studies between* INSIG2* variations and obesity have been performed. The association between rs7566605 and obesity was reported in different populations [3–5], but there were negative reports about this polymorphism and obesity [6–10]. A meta-analysis study declared that rs7566605 was associated with severe obesity (BMI ≥ 32.5, 35.0, 37.5, 40.0) [11]. In the previous study of our research group, the frequency of rs7566605 CC genotype frequency in severe obese group (BMI ≥ 97th percentile) was higher than that of normal-weight group [12]. These evidences showed that the upstream region of* INSIG2* gene might be involved in weight regulation [11, 12].

Krapivner and colleagues [13] screened* INSIG2* gene in search of the functional polymorphism which was responsible for the relationship of rs7566605 and BMI. They discovered a novel variation, -102G>A (rs76335892) in putative promoter region, which was associated with BMI in 1590 middle-aged Caucasian men. In the subsequent research, -102G>A polymorphism was shown to affect the binding of nuclear factor to the promoter region of* INSIG2* [13]. In a following report, Hubáček and colleagues [14] found -102G>A was associated with high-density lipoprotein cholesterol (HDL-C) only in Caucasian female. They did not find the association with BMI either in females or in males. So whether the -102G>A polymorphism is associated with BMI or related phenotypes is not clear. And it is unknown whether the effect is gender-specific, which awaits further investigation.

Considering varied genetic background across populations with different ethnicity, it is necessary to evaluate the effect in non-Caucasian populations. Until now, we did not find any study exploring the relationship between -102G>A polymorphism and obesity in Chinese population. Compared with adults, children have higher BMI or obesity heritability and most obese children are only obese without complications, which help to identify the genetic effects on obesity. In addition, the effect of -102G>A polymorphism has not been studied in children population. Therefore, we performed the present study, aiming to investigate the association of -102G>A polymorphism with obesity-related phenotypes in Chinese children and test gender-specific effects.

## 2. Methods

### 2.1. Subjects

We conducted the study in 2,030 children and adolescents from two independent study groups, including 1,218 boys and 812 girls, 705 obese cases, and 1,325 nonobese controls. The first study group came from the study on adolescent lipids, insulin resistance, and candidate genes (ALIR). The second study group was from the Comprehensive Prevention Project for Overweight and Obese Adolescents (CPOOA) with physical exercise and healthy nutrition as instruments. The ascertainment strategies in two studies have been previously described in detail [12, 15]. All obese individuals in the selected schools were recruited with their voluntary participation. The method of cluster sampling was adopted, to recruit nonobese subjects from some classes of each grade in the same schools. We used the uniform BMI percentile criteria for obese and nonobese children, which were determined in a representative Chinese population [16]. According to the criteria, the children and adolescents with an age- and gender-specific BMI ≥ 95th percentile are defined as obese, whereas those with a BMI between 15th and 95th percentile are nonobese. We chose those with their BMI ≥ 97th percentile as the severely obese group. We excluded the individuals with heart, lung, liver, kidney disease, and cardiovascular or metabolic disease by asking the medical history. Two studies were approved by the Ethic Committee of Peking University Health Science Center. Written informed consent was provided by all participants and, in the case of minors, their parents.

### 2.2. Anthropometric and Biochemical Measurements

Anthropometric measurements, including height, weight, waist, and hip circumference, were determined according to standard protocols [17]. The skinfold thickness on the triceps, subscapula, abdomen, and suprailiumwas measured. The sum of four skinfold thicknesses was calculated as total skinfold thickness. The body fat percentage was determined by bioelectrical impedance analysis (Genius-220, Jawon, Korea). Fasting venous blood samples were taken for measurement of total cholesterol, triglyceride, low-density lipoprotein cholesterol (LDL-C), and high-density lipoprotein cholesterol (HDL-C) using a biochemical autoanalyzer (Hitachi 7060, Tokyo, Japan).

### 2.3. Genotyping

The -102G>A genotype was assayed by using matrix-assisted laser desorption/ionization time-of-flight mass spectrometry (MALDI-TOF MS). Primers, including a pair of amplification primers and an extension primer, were designed with SpectroDESIGNER software (Sequenom, San Diego, CA). A multiplex polymerase chain reaction was performed, and unincorporated double stranded nucleotide triphosphate bases were dephosphorylated with shrimp alkaline phosphatase followed by primer extension. The purified primer extension reaction was spotted onto a 384-element silicon chip (SpectroCHIP, Sequenom) and assayed in MALDI-TOF MS. The resulting spectra were processed with MassArray Typer (Sequenom, San Diego, CA). Our study groups came from two independent studies, that is, ALIR study and CPOOA study. The genotypes of the -102G>A polymorphism were in Hardy-Weinberg Equilibrium (HWE) among nonobese children (*P* = 0.540) of the ALIR study but not in those of the CPOOA study (*P* < 0.001). We performed the genotyping of all the subjects twice by using ARMS-PCR and MALDI-TOF MS, respectively. Except for one sample which was not successfully genotyped by MALDI-TOF MS, the genotyping results were exactly the same for the results of the two genotyping methods. The genotyping concordance rate was 100%. For validity of genotypes, allele assignments were made by at least two experienced individuals independently. Discrepancies were solved unambiguously either by reaching consensus or by repeating. So the deviation from HWE in the CPOOA study was not due to any genotyping error.

### 2.4. Statistical Analyses

The genotype data was tested for deviation from Hardy-Weinberg equilibrium. The differences in general characteristics between obese and nonobese subjects were tested with Chi-square (categorical variables) or *t*-test (continuous variables). The logistic regression model (LRM) was used to examine the effect of -102G>A on obesity. The multiple linear regression (MLR) was performed to examine the effect of -102G>A on BMI or other obesity-related phenotypes (waist and hip circumferences, skinfold thickness, body fat percentage, serum lipids, etc.). LRM and MLR were performed under the dominant model adjusted for gender, age, age square, and study population. All the above analyses were performed with SPSS 18.0 software (SPSS Inc., Chicago, USA). The criterion for statistical significance was set at *P* < 0.05. As the original paper of -102G>A polymorphism just reported its association with BMI (subjects homozygous for the -102A allele had greater decreases in BMI compared with -102G allele carriers [13]), the effect size of the -102G>A polymorphism on risk of obesity was not available. With the assumed OR of 1.4 for obesity, the power for detecting association was estimated to be 84%, given the minor allele frequency (10% in the original paper [13]), the sample size (1325 controls and 705 cases), and the alpha level of 0.05 under the dominant model. Power calculations were performed using the method for gene study of case-control (unmatched) in Quanto software (University of Southern California, Los Angeles, CA).

## 3. Results

### 3.1. General Characteristics of Study Population

The general characteristics of the study groups were shown in Table 1. In total populations, there are more boys in obese group than in nonobese group (68.7% versus 55.4%, *P* < 0.001). The mean age was similar between nonobese group and obese subjects (*P* = 0.497). The levels of BMI, waist circumference, hip circumference, total cholesterol, triglyceride, LDL-C, and skinfold thickness were higher in obese group (*P* ≤ 0.001), while the HDL-C level was lower in obese group, compared with controls (*P* < 0.001). Table 1(b) showed the general characteristics of the study groups in boys or girls. The age difference was not significant between nonobese group and obese subjects in both boys and girls. Except for the total cholesterol levels in girls, the other phenotypes were significantly different between nonobese group and obese subjects in both girls and boys.

### 3.2. Association between -102G>A Polymorphism and Obesity-Related Phenotypes

In all the 2030 samples, 2029 were genotyped successfully for the -102G>A polymorphism. The call rate of the -102G>A polymorphism was 99.95%. There were 1877 (92.5%) children and adolescents being homozygous carriers of G-allele, 142 (7.0%) heterozygous and 10 (0.5%) homozygous carriers of A-allele. The A-allele frequency was 3.99%.

Considering the number of AA genotype carriers was small, we performed the following analyses under dominant genetic model. As shown in Table 2, the percentage of GA/AA genotype carriers in all 2029 subjects was marginally higher in obese group (8.8% versus 6.8%, *P* = 0.063) and significantly higher in severely obese group (10.1% versus 6.8%, *P* = 0.015), compared with the nonobese group. Adjusted for covariates such as gender, age, age square, and study population, the GA/AA genotype was still marginally associated with high risk of obesity (OR = 1.41, 95% CI: 0.998–2.00, *P* = 0.051, Table 2). We found a stronger association between GA/AA genotype and increased risk to be severely obese (OR = 1.62, 95% CI: 1.11–2.36, *P* = 0.012).

Subsequently, we stratified the population by gender and found the GA/AA genotype was significantly associated with risk of severe obesity only in boys (OR = 1.91, 95% CI: 1.15–3.17, *P* = 0.012). The association with increased risk of obesity in boys was marginal (OR = 1.57, 95% CI: 0.996–2.46, *P* = 0.052). We tested for statistical interaction between the SNP and gender on severe obesity. The interaction did not reach statistically significant level (*P* = 0.306).

In addition, we analyzed the relationship between the -102G>A polymorphism and quantitative obesity-related phenotypes. A gender-specific association of -102G>A polymorphism with waist circumference was found in boys (*β* = 2.61 cm, *P* = 0.013, Table 3). We did not find the association between -102G>A polymorphism and waist circumference in girls. In the subsequent analyses we did not find any association of the polymorphism with hip circumference, WHR, lipids levels (total cholesterol, triglyceride, LDL-C, and HDL-C), body fat percentage, or skinfold thickness (*P* > 0.05, Table 3). The results were similar in nonobese participants (*P* > 0.05, data not shown).

## 4. Discussion

To our knowledge, this is the first study to investigate the association between -102G>A polymorphism and obesity-related phenotypes in Chinese children. We found a gender-specific effect of -102G>A polymorphism on severe obesity and waist circumference only in boys.

The -102G>A was found to be associated with BMI in the discovery study [13]. The present data did not support evidence for its association with BMI but showed an association for severe obesity compared to nonobese group (*P* = 0.012). In the previous investigation, the association between* INSIG2* upstream polymorphism rs7566605 and obesity or severe obesity did not reach significant statistic level in our population [12]. The association of -102G>A with severe obesity suggested that the proximal promoter region might impact the function of* INSIG2* gene. Of note, the association for severe obesity was only observed in boys in the further gender-stratified analyses. Additionally, the -102G>A polymorphism was associated with waist circumference and tended to affect hip circumference and WHR levels in boys. Taken together, it appears that the -102G>A polymorphism affects both general and central obesity in Chinese boys.

There are evidences for difference in body composition, hormones, and genetic and environmental factors between boys and girls [18, 19]. It was previously reported that the effects of some obesity-related gene variants have gender-specific effects [20, 21], including a male-specific effect of the near-*INSIG2* variant (rs7566605, upstream -102G>A) on waist-hip ratio in Norway adolescents and adults [21]. In line with the Norway research, the effects of -102G>A polymorphism on severe obesity and waist circumstance in the present study were significant only in boys. It is interesting that two previous reports observed different gender-specific effect of -102G>A polymorphism in European populations [13, 14]. Further investigation is required to examine how gender influences the association between -102G>A polymorphism and obesity-related phenotypes.

In the discovery study, Krapivner and colleagues [13] found -102G-allele was significantly associated with elevated BMI; that is, the GG genotype carriers had the highest BMI compared with GA or AA genotypes carriers. However, no association was found between -102G>A polymorphism and serum lipids. Hubáček and colleagues [14] conducted another study on -102G>A polymorphism in European white people, discovering that -102G>A polymorphism may affect the HDL-C level in young women. HDL-C level of GG homozygotes was higher than that of the A-allele carriers. They did not find similar association in males and older women, which indicated that gender and age may influence the effect of -102G>A polymorphism on serum lipids. The level of HDL-C is usually higher in normal-weight individuals than that in obese group. The results of Hubáček et al. [14] indicated that the GG homozygotes carriers of -102G>A had higher HDL-C levels and A-allele may be a risk factor for obesity. The present study found A-allele is a risk factor for obesity and waist circumference, which was supported by Hubáček et al.'s results. However, the results of Krapivner and colleagues [13] suggested GG genotype is a risk factor for obesity; that is, A-allele is a protective factor for obesity. The inconsistency may be explained by the differences in race, age, and gender of study subjects, demonstrating the value of conducting genetic studies in different ethnic populations.

INSIG2 is an important protein in sterol regulatory element binding protein (SREBP) activation-related lipid regulation pathway. INSIG2 could combine with the complex of SREBP and SREBP cleavage-activating protein (SCAP) to block the transport of SREBPs from endoplasmic reticulum to Golgi apparatus and then affect lipid synthesis [22]. For obesity is associated with lipid metabolism, INSIG2 may influence the development of obesity by regulating lipid synthesis. Krapivner and colleagues [13] conducted a functional study on* INSIG2*. They found the differentiation process of adipocyte is accompanied by increasing expression of* INSIG2*, while the expression of* INSIG2* seems to be associated with -102G>A polymorphism.* INSIG2* mRNA concentrations tended to be lower in GA heterozygotes compared with GG homozygotes [13]. They demonstrated that the -102G>A polymorphism could affect the binding of nuclear factors to the promoter of* INSIG2*, resulting in changes in the expression of* INSIG2* in adipose tissue, ultimately leading to changes in lipid metabolism [13]. Based on Krapivner's functional study, we consider that -102A allele can lead to less expression of* INSIG2*, and theoretically there will be less INSIG2 protein to block the activation of SREBP, ultimately leading to increase of lipid synthesis, which supported -102A allele is a risk factor of obesity.

The strength of the study was that we conducted the study in children. Compared with adults, children have higher BMI or obesity heritability and most obese children are only obese without complications, which help to identify the genetic effects on obesity. Our finding of the association of -102G>A with severe obesity and waist circumference in boys provided evidence for gender disparity of the* INSIG2* gene effects, which will be helpful for identifying genetic factors related to childhood obesity and developing early prevention strategy. Secondly, we provided evidence for gender-specific effect of -102G>A in a Chinese population different from previous studies.

However, the limitation of the present study is that we did not analyze interaction between the polymorphism and environmental factors, such as exercise and diet for limited sample size. And the mechanism underlying the gender-specific effect is unknown, which awaits further functional studies. The third limitation is that the findings of the present study need further replication in other independent population groups. Statistical power analysis revealed that the present study had power around 80% to detect the association of -102G>A polymorphism with severe obesity in total population and boys (76% and 81%, resp.), but the power was lower in girls for lower OR (17%). Another limitation of this study is the limited sample size of the present study. But our results indicated that the OR of -102G>A for obesity in girls is probably lower than that in boys, which need further confirmation by large sample studies.

In conclusion, the study identified a gender-specific effect of* INSIG2* -102G>A polymorphism on risk of severe obesity and waist circumference in Chinese boys. The findings await further functional and large-scaled population studies for validation.

## Figures and Tables

**(a):** 

	All samples		
	Obese group	Nonobese group	*P* value
*N*	705	1325	—
Age (year)	12.85 ± 2.59	12.93 ± 2.72	0.497
Male (%)	484 (68.7)	734 (55.4)	<0.001
Body mass index (BMI)	28.12 ± 3.94	21.53 ± 3.45	<0.001
BMI-SDS	2.49 ± 0.48	0.85 ± 0.94	<0.001
Waist circumference (cm)	88.12 ± 11.27	71.78 ± 9.91	<0.001
Hip circumference (cm)	100.68 ± 11.15	88.75 ± 11.23	<0.001
Waist-hip ratio (WHR)	0.88 ± 0.06	0.81 ± 0.06	<0.001
Total cholesterol (mmol/L)	4.44 ± 0.78	4.22 ± 0.75	<0.001
Triglyceride (mmol/L)	1.17 ± 0.65	0.84 ± 0.35	<0.001
Low-density lipoprotein cholesterol (mmol/L)	2.64 ± 0.75	2.25 ± 0.65	<0.001
High-density lipoprotein cholesterol (mmol/L)	1.09 ± 0.35	1.27 ± 0.44	<0.001
Body fat percentage (%)	27.46 ± 5.61	19.19 ± 6.75	<0.001
Triceps skinfold (mm)	21.60 ± 4.25	14.41 ± 4.65	<0.001
Subscapular skinfold (mm)	22.02 ± 4.69	13.37 ± 5.38	<0.001
Abdominal skinfold (mm)	23.55 ± 5.37	15.47 ± 5.97	<0.001
Suprailiac skinfold (mm)	20.89 ± 4.62	13.25 ± 5.44	<0.001
Sum of skinfolds (mm)	88.06 ± 16.17	56.51 ± 19.73	<0.001

**(b):** 

	Male			Female		
	Obese group	Nonobese group	*P* value	Obese group	Nonobese group	*P* value
*N*	484	734		221	591	
Age (year)	12.9 ± 2.61	13.16 ± 2.68	0.095	12.74 ± 2.55	12.66 ± 2.75	0.678
Body mass index (BMI)	28.33 ± 3.95	22 ± 3.23	<0.001	27.65 ± 3.89	20.96 ± 3.63	<0.001
BMI-SDS	2.6 ± 0.47	1.05 ± 0.91	<0.001	2.24 ± 0.41	0.61 ± 0.92	<0.001
Waist circumference (cm)	90.23 ± 11.13	74.52 ± 9.71	<0.001	83.44 ± 10.13	68.38 ± 9.06	<0.001
Hip circumference (cm)	101.06 ± 11.18	89.67 ± 10.69	<0.001	99.76 ± 11	87.6 ± 11.78	<0.001
Waist-hip ratio (WHR)	0.89 ± 0.05	0.83 ± 0.05	<0.001	0.84 ± 0.06	0.78 ± 0.05	<0.001
Total cholesterol (mmol/L)	4.44 ± 0.74	4.14 ± 0.74	<0.001	4.44 ± 0.85	4.32 ± 0.74	0.057
Triglyceride (mmol/L)	1.18 ± 0.64	0.81 ± 0.34	<0.001	1.13 ± 0.66	0.87 ± 0.35	<0.001
Low-density lipoprotein cholesterol (mmol/L)	2.65 ± 0.75	2.21 ± 0.66	<0.001	2.63 ± 0.76	2.29 ± 0.63	<0.001
High-density lipoprotein cholesterol (mmol/L)	1.09 ± 0.35	1.21 ± 0.45	<0.001	1.11 ± 0.35	1.34 ± 0.43	<0.001
Body fat percentage (%)	25.68 ± 5.34	16.08 ± 6.31	<0.001	31.16 ± 4.16	22.3 ± 5.66	<0.001
Triceps skinfold (mm)	21.58 ± 4.28	13.93 ± 4.63	<0.001	21.64 ± 4.2	14.92 ± 4.62	<0.001
Subscapular skinfold (mm)	21.94 ± 4.82	13.09 ± 5.22	<0.001	22.16 ± 4.42	13.67 ± 5.54	<0.001
Abdominal skinfold (mm)	24.05 ± 5.46	15.51 ± 5.86	<0.001	22.48 ± 5.03	15.43 ± 6.1	<0.001
Suprailiac skinfold (mm)	21.13 ± 4.77	12.98 ± 5.33	<0.001	20.4 ± 4.25	13.53 ± 5.54	<0.001
Sum of skinfolds (mm)	88.71 ± 16.64	55.51 ± 19.17	<0.001	86.69 ± 15.13	57.54 ± 20.27	<0.001

**Table 2:** Association of the *INSIG2* -102G>A polymorphism with obesity.

	Genotype				OR (95% CI)	*P* value
	GG (%)	GA (%)	AA (%)	GA + AA (%)		
Total						
Nonobese	1234 (93.2)	83 (6.3)	7 (0.5)	90 (6.8)		
Obese	643 (91.2)	59 (8.4)	3 (0.4)	62 (8.8)	1.41 (0.998–2.00)	0.051
Severely obese	427 (89.9)	45 (9.5)	3 (0.6)	48 (10.1)	1.62 (1.11–2.36)	**0.012**
Boys						
Nonobese	691 (94.3)	40 (5.5)	2 (0.3)	42 (5.7)		
Obese	442 (91.3)	39 (8.1)	3 (0.6)	42 (8.7)	1.57 (0.996–2.46)	0.052
Severely obese	248 (89.5)	26 (9.4)	3 (1.1)	29 (10.5)	1.91 (1.15–3.17)	**0.012**
Girls						
Nonobese	543 (91.9)	43 (7.3)	5 (0.8)	48 (8.1)		
Obese	201 (91.0)	20 (9.0)	0 (0)	20 (9.0)	1.23 (0.71–2.16)	0.461
Severely obese	179 (90.4)	19 (9.6)	0 (0)	19 (9.6)	1.30 (0.73–2.32)	0.367

*P* value of logistic regression under dominant model adjusted for age, age square, and study population.

**Table 3:** Associations of the -102G>A polymorphism with obesity-related phenotypes in girls and boys.

	Total			Boys			Girls		
	*β*	SE	*P* value	*β*	SE	*P* value	*β*	SE	*P* value
Body mass index (BMI)	0.39	0.33	0.233	0.73	0.45	0.108	0.003	0.47	0.996
BMI-SDS	0.11	0.09	0.207	0.16	0.12	0.173	0.05	0.13	0.687
Waist circumference (cm)	1.11	0.85	0.195	2.61	1.21	**0.031 **	–0.74	1.17	0.527
Hip circumference (cm)	0.76	0.67	0.256	1.65	0.91	0.072	–0.44	0.96	0.649
Waist-hip ratio (WHR)	0.005	0.005	0.346	0.01	0.01	0.073	–0.004	0.01	0.590
Total cholesterol (mmol/L)	0.02	0.06	0.767	0.05	0.08	0.517	–0.03	0.09	0.771
Triglyceride (mmol/L)	0.07	0.04	0.112	0.07	0.06	0.212	0.06	0.06	0.336
Low-density lipoprotein cholesterol (mmol/L)	0.05	0.06	0.428	0.07	0.08	0.355	0.01	0.08	0.897
High-density lipoprotein cholesterol (mmol/L)	0.02	0.02	0.292	0.01	0.03	0.766	0.03	0.03	0.339
Body fat percentage (%)	0.39	0.77	0.617	1.56	1.20	0.196	–0.64	0.96	0.505
Triceps skinfold (mm)	–0.13	0.63	0.839	0.40	0.94	0.672	–0.86	0.82	0.296
Subscapular skinfold (mm)	0.72	0.72	0.313	1.65	1.06	0.121	–0.37	0.94	0.691
Abdominal skinfold (mm)	0.64	0.76	0.404	1.67	1.15	0.148	–0.61	0.95	0.518
Suprailiac skinfold (mm)	0.39	0.70	0.577	1.06	1.06	0.315	–0.53	0.86	0.541
Sum of skinfolds (mm)	1.62	2.63	0.539	4.78	3.95	0.226	–2.37	3.33	0.476

Multiple linear regression was performed under dominant model adjusted for sex, age, age square, and study population in total, while analyses in boys or girls were adjusted for age, age square, and study population. *β*: unstandardized regression coefficients; SE: standard error.
